# Post hoc analysis examining symptom severity reduction and symptom absence during food challenges in individuals who underwent oral immunotherapy for peanut allergy: results from three trials

**DOI:** 10.1186/s13223-023-00757-8

**Published:** 2023-03-13

**Authors:** Katharina Blumchen, Andreas Kleinheinz, Ludger Klimek, Kirsten Beyer, Aikaterini Anagnostou, Christian Vogelberg, Sergejus Butovas, Robert Ryan, David Norval, Stefan Zeitler, George Du Toit

**Affiliations:** 1 Department of Children and Adolescent Medicine, Division of Allergology, Pneumology and Cystic Fibrosis, University Hospital Frankfurt, Goethe University Frankfurt, Frankfurt, Germany; 2 Elbe Kliniken Buxtehude, Buxtehude, Germany; 3 Zentrum für Rhinologie und Allergologie Wiesbaden, Wiesbaden, Germany; 4 Charité—Universitätsmedizin Berlin, Berlin, Germany; 5 Texas Children’s Hospital & Baylor College of Medicine, Houston, TX USA; 6 Department of Pediatric Pulmonology and Allergy, University Hospital Carl Gustav Carus, Technical University of Dresden, Dresden, Germany; 7 Aimmune Therapeutics, a Nestlé Health Science Company, Munich, Germany; 8 Aimmune Therapeutics, a Nestlé Health Science Company, London, UK; 9 Guy’s and St Thomas’ NHS Foundation Trust, London, UK

**Keywords:** Peanut allergy, Oral immunotherapy, Food allergy, Palforzia, Peanut (*Arachis hypogaea*) allergen powder-dnfp, Food challenge

## Abstract

**Purpose:**

Peanut allergy and its current management, involving peanut avoidance and use of rescue medication during instances of accidental exposure, are burdensome to patients and their caregivers and can be a source of stress, uncertainty, and restriction. Physicians may also be frustrated with a lack of effective and safe treatments other than avoidance in the current management of peanut allergy. Efficacy, determined using double-blind, placebo-controlled food challenges (DBPCFCs), of oral immunotherapy with peanut (*Arachis hypogaea*) allergen powder-dnfp (PTAH; Palforzia^®^) was demonstrated versus placebo in children and adolescents aged 4 to 17 years in multiple phase 3 trials; continued benefit of PTAH was shown in a follow-on trial. The DBPCFC is a reproducible, rigorous, and clinically meaningful assessment accepted by regulatory authorities to evaluate the level of tolerance as an endpoint for accidental exposures to peanut in real life. It also provides useful clinical and patient-relevant information, including the amount of peanut protein an individual with peanut allergy can consume without experiencing dose-limiting symptoms, severity of symptoms, and organs affected upon ingestion of peanut protein. We explored symptoms of peanut exposure during DBPCFCs from phase 3 and follow-on trials of PTAH to further characterize treatment efficacy from a perspective relevant to patients, caregivers, and clinicians.

**Methods:**

Symptom data recorded during screening and/or exit DBPCFCs from participants aged 4 to 17 years receiving PTAH or placebo were examined post hoc across three PTAH trials (PALISADE [ARC003], ARC004 [PALISADE follow-on], and ARTEMIS [ARC010]). The maximum peanut protein administered as a single dose during DBPCFCs was 1000 mg (PALISADE and ARTEMIS) and 2000 mg (ARC004). Symptoms were classified by system organ class (SOC) and maximum severity. Endpoints were changes in symptom severity and freedom from symptoms (ie, asymptomatic) during DBPCFC. Relative risk (RR) was calculated for symptom severity by SOC and freedom from symptoms between groups; descriptive statistics were used to summarize all other data.

**Results:**

The risk of any respiratory (RR 0.42 [0.30–0.60], *P* < 0.0001), gastrointestinal (RR 0.34 [0.26–0.44], *P* < 0.0001), cardiovascular/neurological (RR 0.17 [0.08–0.39], *P* < 0.001), or dermatological (RR 0.33 [0.22–0.50], *P* < 0.0001) symptoms was significantly lower in participants treated with PTAH versus placebo upon exposure to peanut at the end of the PALISADE trial (ie, exit DBPCFC). Compared with placebo-treated participants (23.4%), the majority (76.3%) of PTAH-treated participants had no symptoms at the exit DBPCFC when tested at the peanut protein dose not tolerated (ie, reactive dose) during the screening DBPCFC. Significantly higher proportions of PTAH-treated participants were asymptomatic at doses ≤ 100 mg in the exit DBPCFC compared with placebo-treated participants (PALISADE: 69.35% vs 12.10%, RR 5.73 [95% confidence interval (CI) 3.55–9.26]; *P* < 0.0001; ARTEMIS: 67.42% vs 13.95%, RR 4.83 [95% CI 2.28–10.25]; *P* < 0.0001); findings were similar at peanut protein doses ≤ 1000 mg (PALISADE: RR 15.56 [95% CI 5.05–47.94]; *P* < 0.0001; ARTEMIS: RR 34.74 [95% CI 2.19–551.03]; *P* < 0.0001). In ARC004, as the period of PTAH maintenance became longer, greater proportions of participants were asymptomatic at doses of peanut protein ≤ 1000 mg in the exit DBPCFC (from 37.63% after ~ 6 months of maintenance treatment [exit DBPCFC of PALISADE] to 45.54% after ~ 13 months and 58.06% after ~ 20 months of overall PTAH maintenance treatment).

**Conclusions:**

PTAH significantly reduced symptom severity due to exposure to peanut, which is clinically relevant. When exposed to peanut, participants with peanut allergy treated with PTAH rarely had moderate or severe respiratory or cardiovascular/neurological symptoms. Oral immunotherapy with PTAH appears to reduce frequency and severity of allergic reactions in individuals with peanut allergy after accidental exposure to peanut and may enable them and their families to have an improved quality of life.

*Trial registration* ClinicalTrials.gov, NCT02635776, registered 17 December 2015, https://clinicaltrials.gov/ct2/show/NCT02635776?term=AR101&draw=2&rank=7; ClinicalTrials.gov, NCT02993107, registered 08 December 2016, https://clinicaltrials.gov/ct2/show/NCT02993107?term=AR101&draw=2&rank=6; ClinicalTrials.gov, NCT03201003, registered 22 June 2017, https://clinicaltrials.gov/ct2/show/NCT03201003? term = AR101&draw = 2&rank = 9

## Background

Peanut allergy is generally a lifelong condition than can lead to life-threatening reactions and/or substantial symptom burden upon accidental ingestion of peanuts in some individuals [1, 2]. Although the standard of care for peanut allergy to reduce accidental exposure is peanut avoidance, avoidance itself can be a major source of stress, uncertainty, and restrictions [3–6]. Peanut allergy negatively impacts allergy-specific and general quality of life in individuals with peanut allergy and their caregivers [7]. In the Peanut Allergy Burden Study, about two-thirds of participants indicated that their daily living was at least “somewhat” impaired as a result of their peanut allergy; findings from the Allergy to Peanuts imPacting Emotions And Life (APPEAL) surveys showed a majority of respondents were frustrated and many felt anxious/tense, worry, or fear due to living with peanut allergy [7, 8].

Peanut is a common food globally, and the risk of accidental exposure is an important issue [9]. Traces of peanut protein range from 2.8 to 49,000 parts per million (or mg/kg) in packaged nutrition bars with advisories or noted to contain peanut as a minor ingredient [9, 10]. The median amount of peanut protein triggering a reaction is reported to be 125 mg [11]. Patients may underutilize rescue medication in response to an allergic reaction due to lack of knowledge or fear surrounding epinephrine administration, and healthcare providers may underprescribe epinephrine in high-risk patients [12]. Furthermore, caregivers may not recognize signs/symptoms of severe allergic reactions warranting epinephrine administration because of variable presentations [13]. Previously, the lack of effective treatments other than avoidance in the current management of peanut allergy was likely frustrating to some physicians [14]. As a consequence of these shortcomings, patients may be motivated to seek treatment to mitigate or reduce risks associated with their peanut allergy [15].

Among patients and families of children who have peanut allergy, a goal of therapy (particularly those pursuing peanut oral immunotherapy) is to be protected from cross-contamination and to reduce the severity of reactions following an accidental exposure; thus, management with the oral immunotherapy, peanut (*Arachis hypogaea*) allergen powder-dnfp (PTAH; Palforzia^®^), is one strategy [2, 16–18]. The efficacy of PTAH was established versus placebo using double-blind, placebo-controlled food challenges (DBPCFCs) in children/adolescents in multiple phase 3 trials, and the effects of continued maintenance treatment with PTAH were demonstrated in a follow-on trial [19–22]. The DBPCFC is used as the reference standard for the diagnosis of food allergy symptoms and a negative reaction in response to a challenge dose can help rule out food allergy [23]. Results from screening and exit peanut DBPCFCs in the PTAH and placebo groups of these clinical trials provide insight into the extent of desensitization that can be achieved with PTAH oral immunotherapy. Additionally, the DBPCFC provides useful clinical information, including the amount of peanut protein a patient can consume without dose-limiting symptoms and, if symptoms are present, the severity and organs affected [24].

Results from the DBPCFC, including the severity of symptoms and organs affected as well as the percentage of patients remaining asymptomatic (ie, free of symptoms) during the exit DBPCFC in clinical trials, are considered patient-relevant and clinically important information for the clinician [25]. To the best of our knowledge, this post hoc analysis was the first study to specifically focus on characterizing treatment efficacy from different trials from a perspective relevant to patients, caregivers, and clinicians.

## Methods

**Fig. 1:**
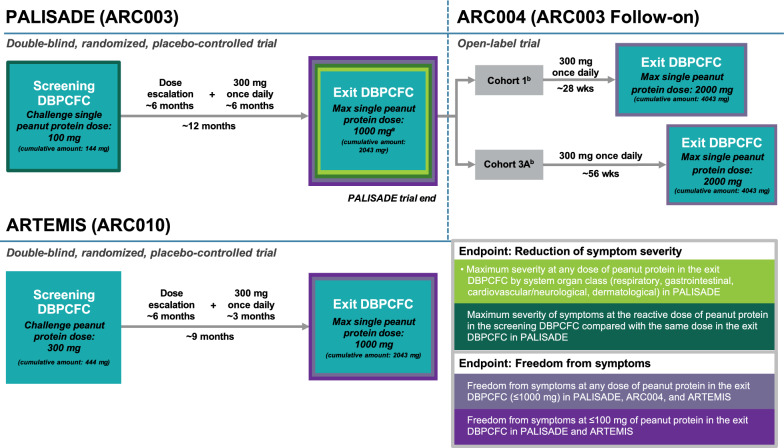
Trial Design for PALISADE, ARC004, and ARTEMIS. Symptom data were recorded from the DBPCFCs across three PTAH trials of participants aged 4 to 17 years who received PTAH or placebo. Endpoints that were examined post hoc focused on evaluating freedom from symptoms and the reduction of symptom severity over time. ^a^The maximum single peanut protein dose tested for the primary clinical efficacy endpoint in PALISADE was 1000 mg in Europe (cumulative amount: 2043 mg) and 600 mg in North America (cumulative amount: 1043 mg). ^b^Following PALISADE, participants treated with PTAH entering ARC004 could be allocated into one of five extended maintenance cohorts. Cohorts 1 and 3A were daily dosing cohorts and are evaluated in this analysis; the nondaily dosing cohorts (2, 3B, 3C) were not included. *DBPCFC* double-blind, placebo-controlled food challenge, *PTAH* peanut (*Arachis hypogaea*) allergen powder-dnfp

Screening and exit DBPCFC data from participants aged 4 to 17 years in the PALISADE (ARC003), ARC004 (PALISADE follow-on), and ARTEMIS (ARC010) trials who received PTAH or placebo were examined post hoc (Fig. 1). Challenge or maximum single peanut protein doses were prespecified for each trial. During the DBPCFCs, peanut protein doses were tested sequentially up to the challenge or maximum single peanut protein dose prespecified for the screening or exit DBPCFC in each trial. To be eligible for trial participation, individuals were required to experience dose-limiting symptoms at or before the prespecified challenge dose of peanut protein in the screening DBPCFC for PALISADE and ARTEMIS. Only participants from PALISADE were eligible for ARC004.

PALISADE was a 12-month, double-blind, randomized, placebo-controlled trial evaluating efficacy and safety of PTAH in individuals with peanut allergy in North America and Europe [21]. A maximum single challenge dose of 100 mg of peanut protein (cumulative dose of 144 mg) was tested in the screening DBPCFC, and the maximum single peanut protein dose tested during the exit DBPCFC was 1000 mg in Europe (cumulative dose of 2043 mg) and 600 mg in North America (cumulative dose of 1043 mg). Participants in PALISADE received ~ 6 months of dose escalation and ~ 6 months of maintenance therapy with PTAH 300 mg or placebo equivalent once daily. To give practical understanding of PTAH therapy in the context of potential real-world exposure, one peanut kernel (of which two are in a usual peanut pod) equates to about 250–300 mg of peanut protein; thus, PTAH 300 mg is equal to approximately one peanut kernel [26].

ARC004 was a 24-month (total duration, including the parent trial duration), open-label, follow-on trial of PALISADE to explore alternate dosing interval regimens and extended maintenance with PTAH [20]. Among the five extended maintenance cohorts enrolled in ARC004 of participants who had received PTAH in PALISADE and continued PTAH therapy in ARC004, only the cohorts receiving daily PTAH (Cohort 1 and Cohort 3A) were included in this post hoc analysis. Participants in Cohort 1 received PTAH 300 mg once daily for ~ 7 months (ie, ~ 13 months overall maintenance treatment), and participants in Cohort 3A received PTAH 300 mg once daily for ~ 14 months (ie, ~ 20 months overall maintenance treatment). A maximum single challenge dose of 2000 mg (cumulative dose of 4043 mg) of peanut protein was tested during the exit DBPCFC in ARC004.

ARTEMIS was a 9-month, double-blind, randomized, placebo-controlled trial evaluating efficacy and safety of PTAH in children and adolescents with peanut allergy in Europe [22]. A maximum single challenge dose of 300 mg peanut protein (cumulative dose of 444 mg) was tested in the screening DBPCFC, and the maximum single dose of peanut protein tested at the exit DBPCFC in ARTEMIS was 1000 mg (cumulative dose of 2043 mg). Participants in ARTEMIS received ~ 6 months of dose escalation followed by ~ 3 months maintenance therapy of PTAH 300 mg or placebo equivalent once daily.

Eligibility criteria varied across the three trials. In PALISADE, eligible participants were between the ages of 4 and 55 years, had clinical history of allergy to peanut or peanut-containing foods, dose-limiting symptoms at or before the 100-mg challenge dose of peanut protein at the screening DBPCFC, and had serum immunoglobulin E (IgE) to peanut of  ≥ 0.35 kUA/L and/or a peanut skin prick test (SPT) wheal diameter  ≥ 3 mm compared with control. Eligible participants who completed PALISADE and gave written informed consent were enrolled in the ARC004 trial. In the ARTEMIS trial, eligible participants were between the ages of 4 and 17 years, had clinical history of allergy to peanut or peanut-containing foods, had dose-limiting symptoms at or before the 300-mg challenge dose of peanut protein at the screening DBPCFC, serum IgE to peanut of  ≥ 0.35 kUA/L and/or a peanut SPT wheal diameter  ≥ 3 mm compared with control. Additional study design details for PALISADE, ARC004, and ARTEMIS have been previously reported [20–22].

**Fig. 2:**
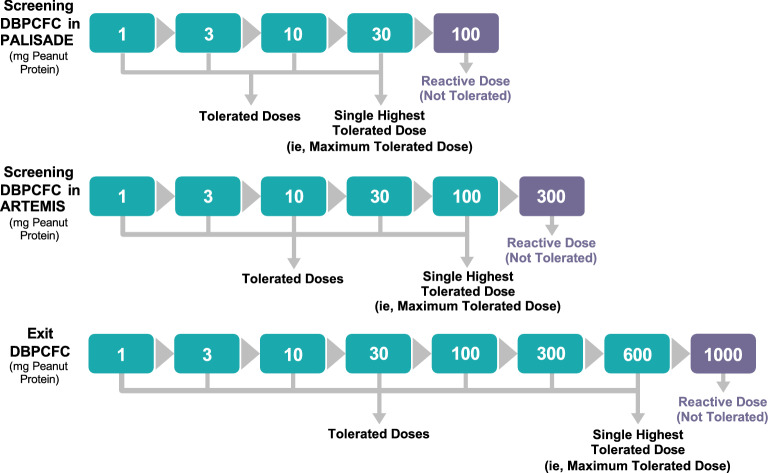
Example to Illustrate the Reactive Dose. Step-up challenge dosing schedule for screening and exit DBPCFC for the PALISADE trial. Participants who were in the ARTEMIS (ARC010) trial had a maximum screening DBPCFC dose of 300 mg. Participants who tolerated doses of peanut protein had subsequently higher doses until a reactive dose was reached. The prior dose to the reactive dose was defined as the single highest tolerated dose, or maximum tolerated dose. *DBPCFC* double-blind, placebo-controlled food challenge

In this analysis, the DBPCFC was used to demonstrate accidental peanut exposure in the real world (ie, simulation of exposure to peanut), and results from the screening and exit DBPCFC were used to provide insight into the amount of peanut protein a participant could safely consume before and after treatment. Endpoints were change in symptom severity and freedom from any symptoms during DBPCFC. The maximum tolerated dose (ie, single highest tolerated dose) was the highest peanut protein dose given during a titrated DBPCFC that elicited no symptoms or symptoms that were not considered dose-limiting (ie, clearly indicative of an allergic reaction; Fig. 2). The reactive dose was the peanut protein dose at which a participant showed dose-limiting symptoms and was one dose level after the maximum tolerated dose.

Change of symptom severity to peanut was assessed using maximum severity at any dose of peanut protein in the exit DBPCFC by system organ class (SOC) in the PALISADE trial and maximum severity of symptoms at the reactive dose of peanut protein in the screening DBPCFC compared with the same dose in the exit DBPCFC in the PALISADE trial. Severity of symptoms presenting during the DBPCFC were assessed using the PRACTALL (ie, dose-limiting symptoms) and the Consortium of Food Allergy Research (CoFAR) grading systems (ie, severity) for allergic reactions during the trial [20–22, 27]. The CoFAR grading system for allergic reactions defines five grades in ascending order of severity: mild (grade 1), moderate (grade 2), severe (grade 3), life-threatening (grade 4), and death (grade 5), each with specific definitions based on timing, limitations in activity, and presenting symptoms [27]. Severity of symptoms was also determined based on investigator’s judgment. Maximum symptom severity was assessed and reported as no symptoms, mild, moderate, or severe, and grouped by the following SOCs: respiratory, gastrointestinal (GI), cardiovascular/neurological, and dermatological. Maximum severity of symptoms was also assessed at the reactive dose in the screening DBPCFC and compared with the same dose in the exit DBPCFC for the PALISADE trial to evaluate dose-dependent changes of symptom severity at a patient-individual dose of peanut protein. For participants who reacted to a lower peanut protein dose during the exit DBPCFC than the screening DBPCFC, data from screening DBPCFC were classified as “with replacement” (WR). The maximum severity of symptoms reported during the screening DBPCFC was used as the exit DBPCFC maximum symptom severity because the reactive dose was lower during the exit DBPCFC than at the screening DBPCFC.

Freedom from any symptoms to peanut was assessed at doses  ≤ 100 mg of peanut protein in the exit DBPCFC in PALISADE and ARTEMIS and at doses  ≤ 1000 mg of peanut protein in the exit DBPCFC in the PALISADE, ARC004, and ARTEMIS trials. Freedom from any symptoms indicated participants were asymptomatic to peanut exposure at the tested peanut protein dose.

### Statistical analyses

Descriptive statistics were reported for each treatment group. Comparisons between treatment groups were conducted using relative risk (RR), which was the primary efficacy measure. The Cochran-Mantel-Haenszel statistic was used to test for treatment difference between screening and exit DBPCFC (if applicable) and difference between treatment groups. Analyses were conducted in the intention-to-treat population, wherein participants who dropped out during the trial were considered “not improved.” For evaluations of maximum symptom severity during DBPCFCs in PALISADE, exit DBPCFC data were analyzed WR, as previously described. Participants reacting to a lower peanut protein dose in the exit DBPCFC compared with the screening DBPCFC only occurred in the placebo group. The WR analysis also reported participants with missing data and dropouts.

## Results

Among the 499 participants who were 4 to 17 years of age randomized to treatment in PALISADE, 374 were assigned to PTAH (372 received at least one dose) and 125 were assigned to placebo (124 received at least one dose); among these participants, 294 (79%) in the PTAH group and 115 (92%) in the placebo group completed the trial. Following completion of the PALISADE trial, 112 participants were enrolled in Cohort 1 and 31 were enrolled in Cohort 3A. Of these participants in the ARC004 trial, 102 (91%) in Cohort 1 and 26 (84%) in Cohort 3A completed the trial. Lastly, in ARTEMIS, among the 175 participants randomized to treatment, 132 were assigned to PTAH and 43 were assigned to placebo. All participants received at least one dose of study treatment. Following ~ 9 months of therapy, 106 (80%) in the PTAH group and 40 (93%) in the placebo group completed the trial (Fig. 1).

**Fig. 3:**
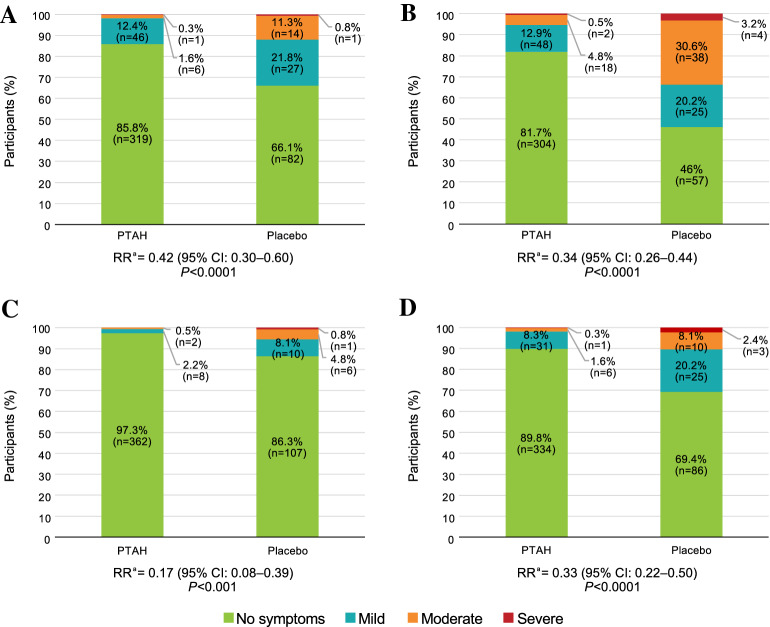
Maximum Symptom Severity at Exit DBPCFC in PALISADE by SOC. **A** Respiratory symptoms. **B** Gastrointestinal symptoms. **C** Cardiovascular/neurological symptoms. **D** Dermatological symptoms. Maximum severity was assessed at any dose of peanut protein in the exit DBPCFC by SOC in the PALISADE trial within the PTAH-treated and the placebo-treated participants. ^a^RR for symptoms of any severity, PTAH vs placebo. *CI* confidence interval, *DBPCFC* double-blind, placebo-controlled food challenge, *PTAH* peanut (*Arachis hypogaea*) allergen powder-dnfp, *RR* relative risk, *SOC* system organ class

The risk of any respiratory, GI, cardiovascular/neurological, or dermatological symptoms was significantly lower in participants treated with PTAH upon exposure to peanut at the end of the PALISADE trial (Fig. 3). Participants who received PTAH versus placebo had an RR of 0.42 (95% confidence interval [CI] 0.30–0.60; *P* < 0.0001) for respiratory symptoms (Fig. 3A), an RR of 0.34 (95% CI 0.26–0.44; *P* < 0.0001) for GI symptoms (Fig. 3B), an RR of 0.17 (95% CI 0.08–0.39; *P* < 0.001) for cardiovascular/neurological symptoms (Fig. 3C), and an RR of 0.33 (95% CI 0.22–0.50; *P* < 0.0001) for dermatological symptoms (Fig. 3D). Participants receiving PTAH versus placebo had lower proportions of severe symptoms across SOCs at the exit DBPCFC.

Compared with placebo-treated participants (n = 29, 23.4%), the majority of PTAH-treated participants (n = 284, 76.3%) had no symptoms in the exit DBPCFC at the reactive dose in the screening DBPCFC within the PALISADE trial (Table 1). No PTAH-treated participants had severe symptoms in the exit DBPCFC. Dose-dependent reductions in symptom severity at a participant-specific dose of peanut protein were observed.

**Table 1:** Maximum Symptom Severity at Reactive Doses During Screening and Exit DBPCFC in PALISADE

Maximum symptom severity (Grade)	PTAH		Placebo	
	Screening DBPCFC^a^ n (%)	Exit (WR^b^) DBPCFC n (%)	Screening DBPCFC^a^ n (%)	Exit (WR^b^) DBPCFC n (%)
None (0)	0 (0)	284 (76.3)	0 (0)	29 (23.4)
Mild (1)	109 (29.3)	11 (3)	35 (28.2)	44 (35.5)
Moderate (2)	216 (58.1)	1 (0.3)	80 (64.5)	38 (30.6)
Severe (3)	47 (12.6)	0 (0)	9 (7.3)	5 (4)
Missing	0 (0)	76 (20.4)	0 (0)	8 (6.5)
Total	372 (100)	372 (100)	124 (100)	124 (100)

^a^Participants were required to experience dose-limiting symptoms at or before 100 mg

^b^WR, or “with replacement,” indicates participants who reacted to a lower dose during the exit DBPCFC compared with the screening DBPCFC and assigned with their maximum symptom severity during the screening DBPCFC reactive dose—this only occurred in the placebo group. The exit WR analysis also reports missing data and dropouts; the analysis does not replace missing data or dropouts

*DBPCFC* double-blind, placebo-controlled food challenge, *PTAH* peanut (*Arachis hypogaea*) allergen powder-dnfp, *WR* with replacement

**Fig. 4:**
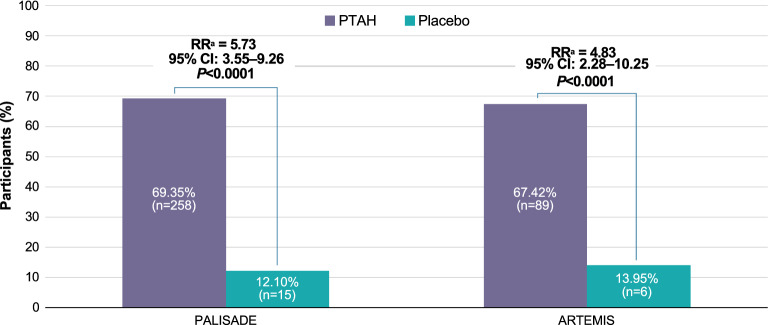
Participants Asymptomatic at Peanut Protein Doses  ≤ 100 mg in the Exit DBPCFC. Freedom from symptoms up to 100 mg of peanut protein in randomized, controlled trials only. Freedom from symptoms indicated participants were asymptomatic to peanut exposure during the entire DBPCFC. ^a^RR (PTAH vs placebo) for any symptoms in the intention-to-treat population. *CI* confidence interval, *DBPCFC* double-blind, placebo-controlled food challenge, *PTAH* peanut (*Arachis hypogaea*) allergen powder-dnfp, *RR* relative risk

**Fig. 5:**
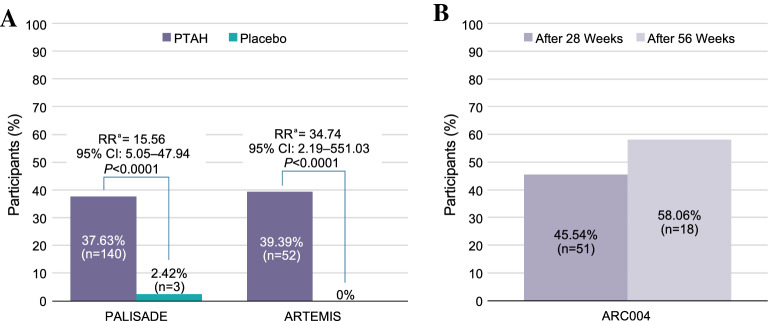
Participants Asymptomatic at All Peanut Protein Doses  ≤ 1000 mg in the Exit DBPCFC. **A** In randomized, controlled trials. **B** In the open-label, follow-on trial. Freedom from symptoms up to 1000 mg of peanut protein. Freedom from symptoms indicated participants were asymptomatic to peanut exposure during the entire DBPCFC. ^a^RR (PTAH vs placebo) for any symptoms in the intention-to-treat population. *CI* confidence interval, *DBPCFC* double-blind, placebo-controlled food challenge, *PTAH* peanut (*Arachis hypogaea*) allergen powder-dnfp, *RR* relative risk

In PALISADE and ARTEMIS, significantly higher proportions of PTAH-treated participants were asymptomatic upon exposure to low doses (≤ 100 mg) of peanut protein in the exit DBPCFC compared with placebo-treated participants (PALISADE: 69.35% vs 12.10%, RR 5.73 [95% CI 3.55–9.26]; *P* < 0.0001; ARTEMIS: 67.42% vs 13.95%, RR 4.83 [95% CI 2.28–10.25]; *P* < 0.0001; Fig. 4). At all doses ≤ 1000 mg, significantly higher proportions of PTAH-treated participants completed the exit DBPCFC without reporting any symptoms compared with placebo-treated participants in PALISADE (37.63% [n = 140] vs 2.42% [n = 3], RR 15.56 [95% CI 5.05–47.94]; *P* < 0.0001) after ~ 6 months of overall PTAH maintenance, and ARTEMIS (39.39% [n = 52] vs 0%, RR 34.74 [95% CI 2.19–551.03]; *P* < 0.0001; Fig. 5A) after ~ 3 months of overall PTAH maintenance. Within ARC004, the proportion of participants with no symptoms in a simulation of accidental exposure to peanut (given doses of peanut protein ≤ 1000 mg) with longer maintenance treatment was numerically higher at 45.54% (n = 51) after ~ 7 months of ongoing maintenance treatment (~ 13 months total of PTAH maintenance treatment) and 58.06% (n = 18) after ~ 14 months of ongoing maintenance treatment (~ 20 months total of maintenance treatment; Fig. 5B).

## Discussion

Overall, the severity and frequency of symptoms presenting upon controlled peanut exposure (ie, the DBPCFC) were significantly (*P* < 0.0001) reduced in participants who received PTAH compared with those who received placebo. PTAH-treated participants exposed to peanut had reduced rates of moderate and severe cardiovascular/neurological symptoms, and respiratory symptoms were almost absent; reduced symptom severity was observed across multiple organ systems. Participants who received PTAH were 2.38 times less likely to suffer from respiratory symptoms, 2.96 times less likely to suffer from GI symptoms, 5.75 times less likely to suffer from cardiovascular/neurological symptoms, and 3.00 times less likely to suffer from dermatological symptoms compared with participants who received placebo.

Cardiovascular/neurological (eg, confusion or unconsciousness) and respiratory (eg, dyspnea or wheezing) symptoms may indicate conditions that increase the risk of morbidity and mortality acutely and therefore be of greater relevance to patients, caregivers, and healthcare providers when evaluating the efficacy of treatments for peanut allergy [28, 29]. One study found that children with allergic diseases associated with respiratory issues, such as wheezing and asthma, had reduced health-related quality of life [30]. PTAH not only reduced the proportion of individuals with severe symptoms across all symptoms by SOC, rates of mild and moderate reactions observed during screening DBPCFC were also reduced and the majority of PTAH-treated participants had no symptoms to peanut exposure by the end of the PALISADE trial. These data provide perspective on what can be expected from PTAH treatment (and subsequent planning of mitigation strategies) and are likely reassuring to individuals with peanut allergy and/or their caregivers, as well as clinicians.

Significantly higher proportions of participants treated with PTAH were asymptomatic during DBPCFC versus those treated with placebo across trials, regardless of maximum peanut dose ingested. Compared with the proportion of PTAH-treated participants in the PALISADE and ARTEMIS trials who could tolerate peanut protein without dose-limiting symptoms (ie, mild symptoms permitted), the proportion of participants who were asymptomatic to peanut protein in this analysis were lower, owing to differences in defining endpoints from a clinical trial versus patient-relevant perspective. Considering that a median dose of 125 mg of peanut protein has been reported to be an estimate for the amount of peanut protein that will trigger an allergic reaction, PTAH-treated participants were identified to be 4.83 to 5.73 times more likely to tolerate a dose of at least 100 mg of peanut protein without symptoms, and thus, potentially less likely to react upon accidental exposure to low quantities of peanut [11]. Participants receiving PTAH in PALISADE and ARTEMIS were also 15.56 and 34.74 times, respectively, more likely to tolerate peanut protein exposure up to 1000 mg and remain asymptomatic versus participants receiving placebo. Of note, all participants in the PALISADE trial were required to experience dose-limiting symptoms at or before 100 mg, which explains why the number of participants with no symptoms is zero in PTAH and placebo groups in the screening DBPCFC. Improvement in symptom freedom was also seen as participants remained on PTAH maintenance therapy over time. In PALISADE, 37.63% of participants who received ~ 6 months of PTAH maintenance were asymptomatic at the exit DBPCFC trial. As this population of participants entered into ARC004, rates of asymptomatic participants further improved. Over 45% of participants tolerated up to 1000 mg of peanut protein without symptoms at ~ 13 months of PTAH maintenance, and rates improved even further at ~ 20 months (58.1%) during the exit DBPCFC. This finding supports that longer maintenance treatment provided continued protection from allergic symptoms in response to peanut exposure.

Overall, more granular assessment of changes in symptoms and symptom presentation at the end of treatment between PTAH- and placebo-treated participants in trials supports primary trial findings and provides added clinical context for patients, caregivers, and healthcare providers. PALISADE showed ~ 67% of children and adolescents with peanut allergy in the PTAH group could tolerate at least 600 mg of peanut protein (approximately two whole peanut kernels) during the exit DBPCFC [21]; ARC004 demonstrated that continued daily treatment with PTAH resulted in continued efficacy [20]. ARTEMIS showed that the median single highest dose tolerated by children with peanut allergy increased 100 times in more than half the participants (58%) who received PTAH compared with placebo (2%) [22]. Although regulatory agencies interested in capturing efficacy and safety of an intervention accurately in a clinical trial setting have provided guidance for use of food challenges to objectively assess the degree of desensitization, these practices may not always apply or be used in the clinical setting [31]. What may be more relevant to individuals with peanut allergy and their caregivers than the degree of desensitization achieved from PTAH to a specific dose of peanut protein may be an understanding of how likely it is for PTAH to provide protection against accidental peanut exposure (ie, remain asymptomatic with exposure) and to what degree symptoms (and type of symptoms) might be expected upon accidental exposure. Addressing concerns related to accidental peanut exposure can subsequently lead to improvements in health-related quality of life, particularly behaviors (although individuals taking PTAH must continue a peanut-avoidant diet) and emotions (ie, fear, anxiety, and worry) associated with avoiding severe and potentially fatal allergic reactions [17, 22, 32].

Several limitations should be considered. Both subjective (ie, abdominal discomfort/pain, nausea, and headache) and objective (ie, changes in spirometry and hypotension) symptoms were included in this analysis, and analyses of symptoms by participant subjectivity (ie, symptoms) or objectivity (ie, signs) could not be clearly differentiated. The type of symptoms reported by participants could also vary over time (eg, from GI to dermatological symptoms). The analysis did not differentiate symptoms within an organ system, such as upper versus lower respiratory symptoms. Analyses of relationships, such as correlations or associations between the level of desensitization after peanut exposure and subsequent symptom presentation, were not performed. Since these trials required participants to undergo a screening DBPCFC prior to enrollment, further study may be warranted in this area to identify specific outcomes and evaluate real-world efficacy where patients are unlikely to undergo oral food challenges prior to starting oral immunotherapy. Lastly, the number of participants with longer-term maintenance treatment in the ARC004 data analysis was small (ie, N = 31 for patients with 56 weeks of open-label maintenance treatment in Cohort 3A); further study may be warranted in a larger sample size to better distinguish the results of long-term PTAH maintenance therapy.

In conclusion, PTAH demonstrated dose-independent and dose-dependent reduction of symptom severity after exposure to peanut. When exposed to peanut, participants treated with PTAH rarely had moderate or severe respiratory or cardiovascular/neurological symptoms. More than one-third of the participants showed no symptoms upon exposure of up to 1000 mg of peanut protein after treatment with PTAH in comparison to none of the placebo-treated participants. Oral immunotherapy with PTAH seems to reduce the risk and severity of allergic reactions after accidental exposure to peanut in individuals with peanut allergy and may enable them and their families to have an improved quality of life. We believe that this is the first analysis to specifically focus on characterizing treatment efficacy from the perspective of patients, caregivers, and clinicians.

## Data Availability

All relevant data are within the manuscript. Clarification requests around the manuscript and its data can be made to the corresponding author.

## Abbreviations

CI: Confidence interval
CoFAR: Consortium of Food Allergy Research
DBPCFC: Double-blind, placebo-controlled food challenge
GI: Gastrointestinal
PTAH: Peanut (*Arachis hypogaea*) allergen powder-dnfp
RR: Relative risk
SOC: System organ class
WR: With replacement
